# Prise en charge chirurgicale des méningiomes intracrâniens à Nouakchott, Mauritanie

**DOI:** 10.11604/pamj.2018.31.146.16485

**Published:** 2018-10-26

**Authors:** Ahmed-Salem Kleib, Brahim Hamad Ngaidé, Ahmedou El Mokhtar Eleit, Seck Mame Diack, Sidi Salem-Memou, Sidi-Mohamed Salihy, Outouma Soumaré

**Affiliations:** 1 Faculté de Médecine, Université de Nouakchott Al-Aasriya, Nouakchott, Mauritanie; 2 Department of Neurosurgery, CHRU Bretonneau, Tours, France; 3 Centre Hospitalier National, Nouakchott, Mauritanie

**Keywords:** Méningiomes, epidémiologie, mauritanie, Meningiomas, epidemiology, Mauritania

## Abstract

Les méningiomes constituent une pathologie couramment rencontrée en neurochirurgie, pourtant les données sur leur épidémiologie, leurs caractéristiques cliniques et leur prise en charge thérapeutique restent rares par rapport aux gliomes. Notre but est de dégager un profil épidémiologique et d'évaluer la qualité de la prise en charge des méningiomes intracrâniens (MIC) au CHN de Nouakchott, Mauritanie. Nous avons revu dans cette étude rétrospective, les observations des patients opérés d'un MIC, entre septembre 2013 et septembre 2016. Trente-deux patients ont été opérés d'un MIC (26,6%). L'âge moyen était de 45,12 ans (± 13,8 ans) dont 75% étaient des femmes. La durée moyenne d'hospitalisation était de 13 jours (± 7jours). Le délai avant le diagnostic était en moyenne de 10 mois (± 5mois). La taille moyenne des MIC était de 5,07 cm (± 2,00cm) avec des extrêmes allant de 2,5cm à 10,5cm. L'IRM a été réalisée chez 46,8% en complément de la TDM cérébrale. Dans notre série 38% des MIC sont localisés sur la convexité. Le délai d'attente opératoire était de 23.91 jours (±17jours). La qualité d'exérèse chirurgicale selon le score de Simpson était de Grade I (66%), Grade II: (19%), Grade III: (6%), Grade IV: (9%). Le type histologique de la classification de l'OMS 2007 était de Garde I (93%), Grade II-III (7%). La mortalité opératoire globale était de n=3 ; 9,4%. Le perfectionnement des plateaux techniques dans le service de neurochirurgie, de radiologie et d’anesthésie-réanimation prochainement va contribuer à l'amélioration de nos résultats et à la diminution de notre taux de mortalité.

## Abstract (en)

Meningiomas are frequently encountered tumours in neurosurgery. However, there is a paucity of data concerning their epidemiology, their clinical characteristics and their treatment compared to gliomas. This study aims to identify the epidemiological profile and to assess the quality of treatment of intracranial meningiomas (ICM) at the National Hospital Center in Nouakchott, Mauritania. We conducted a retrospective study of patients who had undergone surgery for the treatment of ICM between September 2013 and September 2016. Thirty-two patients had undergone surgery for the treatment of ICM (26.6%). The average age was 45.12 (± 13.8 years) among whom 75% were women. The average length of stay in hospital had been 13 days (± 7 days). The mean time between symptom onset and diagnosis had been 10 months (±5months). The mean size of ICM had been 5.07cm (±2.00cm) ranging from 2.5cm to 10.5cm. Complementary MRI had been performed in 46.8% of patients after brain CT scan. In our series, 38% of ICM had grown on brain convexity. The mean surgical time had been 23.91 days (±17days). The quality grade of surgical resection assigned according to Simpson score was Grade I (66%), Grade II (19%), Grade III (6%), Grade IV (9%). The histological grade assigned according to the 2007 WHO classification was Grade I (93%), Grade II-III (7%). The overall operative mortality was (n=3, 9.4%). The development of technical equipment in the department of neurosurgery, radiology and of equipment in anesthesia and intensive care will contribute to improve outcomes and to reduce mortality rates.

## Introduction

Les méningiomes représentent 20 à 30 % des tumeurs intracrâniennes. Leur incidence augmente avec l'âge et touche plus préférentiellement la population noire que blanche [1-3]. Ils sont localisés, le plus souvent, sur la voûte crânienne et la base du crâne, mais ils peuvent également être situés dans la moelle épinière [4]. Les méningiomes constituent une pathologie couramment rencontrée en neurochirurgie, pourtant les données sur leur épidémiologie, leurs caractéristiques cliniques et leur prise en charge thérapeutique restent rares par rapport aux gliomes [4]. En effet, il y a peu d'études africaines sur les méningiomes intracrâniens [5-10]. L'objectif de notre étude est de dégager un profil épidémiologique et d'évaluer la qualité de la prise en charge chirurgicale des méningiomes intracrâniens (MIC) au Centre Hospitalier National (CHN) de Nouakchott, Mauritanie.

## Méthodes

Nous avons revu dans cette étude rétrospective, les observations cliniques, des patients hospitalisés pour prise en charge d'un MIC, entre septembre 2013 et septembre 2016, au service de neurochirurgie du CHN. Nous avons relevé à partir des dossiers des patients opérés d'un MIC, l'âge des patients, leur sexe, la durée d'hospitalisation, le mode de révélation, l'état fonctionnel du patient, la qualité de l'exérèse chirurgicale et le résultat anatomopathologique. En ce qui concerne le suivi à long terme des patients, nous avons noté le recul à la date de la dernière consultation. Les données ont été traitées sur le logiciel SPSS-20 d'IBM.

## Résultats

**Figure 1:**
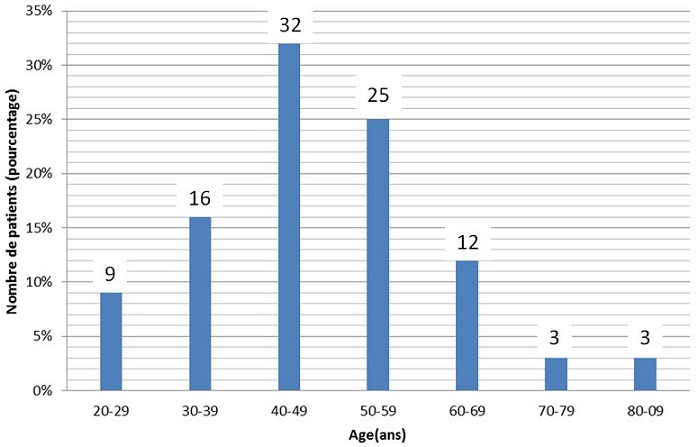
répartition selon l’âge

**Figure 2:**
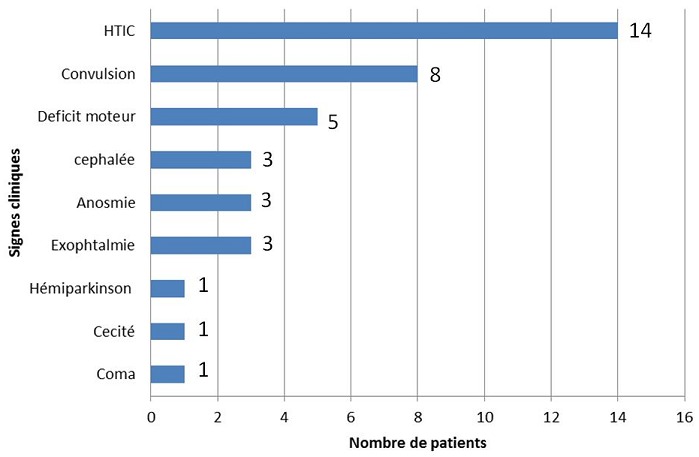
répartition des patients selon le motif d’hospitalisation

**Figure 3:**
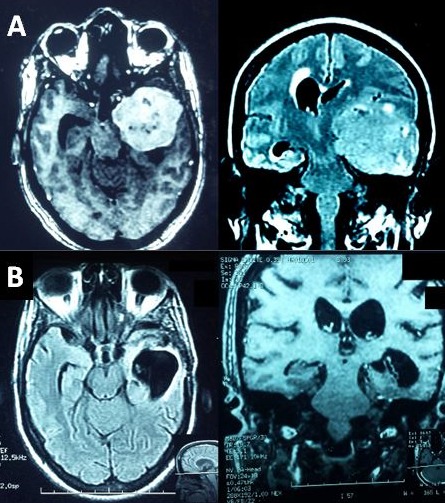
A) IRM cérébrale avec injection de gadolinium, qui a montré un volumineux méningiome, de l’arête sphénoïdale gauche, avec un œdème péri-lésionnel important; B) IRM cérébrale avec injection de gadolinium postopératoire

Trente-deux patients ont été opérés d'un MIC sur 120 patients hospitalisés porteurs d'une tumeur cérébrale durant la durée de l'étude (3 ans) soit un taux de 26,6%. L'âge moyen de nos patients était de 45,12 ans (±13,8 ans) avec une médiane de 46 et des extrêmes allant de 16 à 80 ans (Figure 1). Parmi nos patients 75% étaient des femmes, soit un ratio homme-femme égal à 1/3. La durée moyenne d'hospitalisation était de 13 jours (±7jours). Le délai avant le diagnostic était en moyenne de 10 mois (±5mois), avec des extrêmes allant de 10 jours à 4 ans. La symptomatologie clinique révélatrice était variable (Figure 2). L'indice fonctionnel de Karnofsky est entre 80-100 (n=22; 69%) entre 50-80(n=10; 31%). La tomodensitométrie (TDM) a été réalisée, chez tous nos patients. La TDM a constitué l'unique bilan chez 17 patients (53%). La taille moyenne de MIC était de 5,07 cm (±2,00cm) avec des extrêmes allant de 2,5cm à 10,5cm. La répartition des MIC est variable selon le siège (Tableau 1). L'imagerie par résonnance magnétique (IRM) a été réalisée chez 15 patients (46,8%) en complément de la TDM cérébrale, dans le cadre du bilan préopératoire. Le délai d'attente opératoire entre la consultation de pré anesthésie et la chirurgie est de 23.91 jours (±7jours). La voie d'abord chirurgicale a été choisie en fonction de la topographie du MIC, de son extension et de nos habitudes chirurgicales. La qualité d'exérèse chirurgicale selon le score de Simpson était de Grade I (n=21 ; 66%), Grade II: (n=6;19%), Grade III: (n=2;6%), Grade IV: (n=3;9%). Les résultats anatomopathologiques classés selon le type histologique de la classification de l'OMS 2007 étaient de, Garde I (n=30;93%), Grade II-III (n=2;7%). La mortalité opératoire globale des méningiomes intracrâniens opérés était de (n=3 ; 9,4%). Les causes de décès étaient l’œdème cérébral. Les autres patients (n=28;87,5%) ont été suivis à 6 mois et à un an après l'intervention. On n'a pas retrouvé de notion de récidive. Le méningiome sphéno-orbitaire (MSO) a nécessité une reprise chirurgicale pour persistance d'exophtalmie. L'hémi-parkinsonisme à l'origine de la découverte d'un méningiome sphénoïdale chez une de nos patientes a disparu après la chirurgie (Figure 3). Cinq patients sont toujours sous antiépileptiques à un an après la chirurgie.

**Tableau 1:** répartition topographique des MIC dans notre série

Localisation		Nombre de cas	Pourcentage
Convexité		12	38
Base du crane			
	Olfactif	5	15
	Ptérional	2	6
	Sphénoïdal	3	9
	Sellaire	1	3
Fosse postérieure		3	9
Para sagittal		6	6

## Discussion

Les méningiomes sont les tumeurs intracrâniennes fréquentes et représentent 35% des tumeurs cérébrales chez l'adulte. L'incidence des méningiomes augmente avec l'âge et touche préférentiellement la population noire que blanche [2, 11]. De Monte *et al.* [12] trouvent une fréquence assez élevée de cette lésion chez les mélanodermes par rapport aux caucasiens, soit une moyenne de 31,1% à travers les études africaines, pour 24% dans les études européennes [12]. Dans notre série, la fréquence était de 26,6%. Cette fréquence est proche de celle observée chez des nombreux auteurs. Mezue *et al.* [6], N'diri *et al.* [8] et Ibebuike *et al.* [13] ont retrouvé respectivement 23,8%, 33,43% et 33,8% de toutes les tumeurs cérébrales primaires. Les auteurs rapportent une prédominance féminine [14], ce qui concorde avec notre série où, 75% de nos patients opérés d'un MIC étaient des femmes. Ce qui est le cas pour Mezue *et al.* [6], qui retrouvaient un rapport hommes/femmes de 1/1,1. Thiam *et al.*, au Sénégal [10], rapportaient une prédominance de 56% de sexe féminin avec un sex-ratio de 0,76 de même pour N'diri *et al.* [8] et Ibebuike *et al.* [13], qui rapportaient respectivement, 57% de sexe féminin avec un sex-ratio de 3/2 et 79.2%. Le diagnostic des méningiomes se fait habituellement entre 20 et 60 ans avec un pic d'incidence à la 5^ème^ décennie [13, 15, 16]. Dans notre série, l'âge moyen était de 45,12 ans (±13,8 ans) avec des extrêmes allant de 16 à 80 ans. La tranche d'âge la plus touchée est située entre 40 et 49 ans avec une fréquence de 32%, ce qui correspond aux données de la littérature. Thiam *et al.*, [10] au Sénégal sur un total de 50 patients opérés d'un MIC, trouvaient une moyenne d'âge de 47,3 ans. L'âge moyen des patients était de 45,7 ± 10,5 ans chez Ibebuike *et al.* [13]. La fréquence la plus élevée était parmi les Africains noirs (75%) et l'ethnie Sotho (27,1%). Dans notre série et dans les séries de la sous-région, nous avons retrouvé la prédominance de la localisation des MIC, au niveau de la convexité cérébrale. Cette localisation représentait pour Sakho et al [5] 50% et Odebode *et al.* [17] 50%, Thiam *et al.*[10] 48%, Duntze *et al.* [18] 60%, Mezue *et al.* [6] 23,5%, et N'diri *et al.* [8] 36%. L'appréciation du délai diagnostic est souvent inexacte, en raison de la difficulté rencontrée à fixer avec précision le début des manifestations cliniques. Dans notre série, le délai moyen de 10 mois avec des extrêmes allant de 10 jours à 4 ans, que nous avons trouvé témoigne du caractère lent et progressif de l'apparition des premiers signes cliniques.

Badiane *et al.* (12) sur une série de 79 cas notaient une durée moyenne d'évolution de 15 mois. Pour N'diri *et al.* [8], Le diagnostic a été fait en moyenne à 22 mois après le début des signes cliniques. Dans notre série, les céphalées représentaient le maître symptôme, (18,75%) et hypertension intracrânienne (HTIC) (43,75%) de même que pour N'diri *et al.* où la symptomatologie clinique était dominée par des céphalées isolées (68,42%) et une HTIC (43,75%) [8]. Les céphalées peuvent être causées par une pression intracrânienne élevée résultant d'une hydrocéphalie, d'un effet de masse de la tumeur elle-même ou d'une hémorragie dans ou autour de la tumeur [19]. Ces céphalées sont probablement autant dues à l'innervation même de la dure-mère, qu'à l'HTIC. Dans notre série la comitialité a été retrouvée dans 25% des cas et a nécessité une exploration par imagerie qui a permis de découvrir la tumeur intracrânienne et extra-axiale. Chez Sakho *et al.* [5] la comitialité était révélatrice dans 37% des cas, et dans 42,10% chez N'diri *et al.* [8]. Englot *et al.* dans une étude méta-analyse trouvaient que des crises préopératoires ont été observées chez 29,2% des 4709 patients atteints de méningiome supratentoriel, et étaient significativement prédites par le sexe masculin (OR 1,74, IC 95% 1,30-2,34); une absence de maux de tête (OR 1,77, IC 95% 1,04-3,25); œdème péritumoral (OR 7,48, IC à 95% 6,13-9,47) une localisation autre que la base du crâne (OR 1,77, IC à 95% 1,04-3,25). Après la chirurgie, 69,3% des 703 patients atteints d'épilepsie préopératoire ont vu les crises cédées. Sur 1085 individus sans épilepsie préopératoire ayant subi une résection de MIC, des nouvelles convulsions postopératoires ont été observées chez 12,3% des patients. Aucune différence dans le taux de nouvelles crises postopératoires n'a été observée avec ou sans anticonvulsivants prophylactiques péri opératoire [20]. Le déficit moteur était retrouvé dans 70% des cas dans l'étude de Sakho *et al.* [5] et 15,62% dans notre série. Les troubles visuels étaient la cécité 3,12% et l'exophtalmie 9,37%, car les patients consultaient tardivement, surtout ceux habitant l'intérieur du pays. Les tumeurs intracrâniennes sont une cause rare de parkinsonisme secondaire [21-23]. La TDM représente un moyen diagnostique fiable des méningiomes, puisque selon Osborn [24] le scanner sans contraste détecte 85% des méningiomes et 95% après injection iodée. Dans notre série ; la TDM a permis de faire le diagnostic de méningiome dans 90% des cas. Dans notre contexte, l'IRM n'est pas indispensable quand les données de la tomodensitométrie sont suffisantes. Toutefois, cet examen présente un intérêt complémentaire car l'IRM reste la technique de référence dans l'exploration des tumeurs de la base. Chez les patients ayant des méningiomes symptomatiques accessibles à la chirurgie, l'objectif est l'exérèse chirurgicale totale [25, 26]. La qualité de la résection chirurgicale est déterminée par une imagerie cérébrale précoce postopératoire. Dans notre série, nous avons contrôlé la qualité de la résection par une TDM dans les 24 heures postopératoires, par sa disponibilité et son faible cout financier. Une corticothérapie à 48H avant l'intervention ou plus, surtout s'il existe un considérable œdème péri lésionnel. Les anti comitiaux ont été poursuivis immédiatement après l'intervention, ou introduits pendant ou au décours de l'intervention. Dans notre série, le type histologique le plus fréquemment rencontré est le type I OMS. L'âge et l'état clinique du patient lors de la prise en charge sont des facteurs pronostiques, universellement reconnus en pratique oncologique et doivent entrer en compte dans la décision thérapeutique. Dans la littérature les seuils de mauvais pronostic sont un âge supérieur à 70 ans et un indice de Karnofsky inférieur à 70 [27-30].

## Conclusion

La bénignité des méningiomes fait de l'exérèse chirurgicale, une obligation thérapeutique. La qualité de survie dépend de l'état clinique préopératoire, de la localisation et de la taille tumorale. La prise en charge des MIC reste insuffisante, malgré les efforts déployés en matière de santé publique et la précocité du diagnostic. L'amélioration de nos résultats et la diminution de la mortalité seront significatives avec le perfectionnement des plateaux techniques dans le service de neurochirurgie, de radiologie et d'anesthésie-réanimation.

### Etat des connaissances actuelles sur le sujet

- Les méningiomes constituent une pathologie couramment rencontrée en neurochirurgie;
- Le diagnostic des méningiomes se fait habituellement entre 20 et 60 ans avec un pic d'incidence à la 5ème décennie;
- La bénignité des méningiomes fait de l'exérèse chirurgicale, une obligation thérapeutique.

### Contribution de notre étude à la connaissance

- Il s'agit de la première étude en Mauritanie sur le sujet;
- Elle nous a permis de dégager un profil épidémiologique au Centre Hospitalier de Nouakchott, Mauritanie, le seul centre du pays;
- Elle nous a permis d'évaluer la qualité de la prise en charge des méningiomes intracrâniens (MIC) en Mauritanie.

## Conflits des intérêts

Les auteurs ne déclarent aucun conflit d'intérêts.
